# APC loss in breast cancer leads to doxorubicin resistance via STAT3 activation

**DOI:** 10.18632/oncotarget.22263

**Published:** 2017-11-01

**Authors:** Monica K. VanKlompenberg, Emily Leyden, Anne H. Arnason, Jian-Ting Zhang, Casey D. Stefanski, Jenifer R. Prosperi

**Affiliations:** ^1^ Department of Biochemistry and Molecular Biology, Indiana University School of Medicine–South Bend, South Bend, IN, USA; ^2^ Harper Cancer Research Institute, South Bend, IN, USA; ^3^ Department of Biological Sciences, University of Notre Dame, Notre Dame, IN, USA; ^4^ Department of Pharmacology and Toxicology, Indiana University School of Medicine, Indianapolis, IN, USA; ^5^ Current address: Department of Animal and Avian Sciences, University of Maryland, College Park, MD, USA

**Keywords:** Adenomatous Polyposis Coli, breast cancer, STAT3, chemoresistance, doxorubicin

## Abstract

Resistance to chemotherapy is one of the leading causes of death from breast cancer. We recently established that loss of *Adenomatous Polyposis Coli* (APC) in the Mouse Mammary Tumor Virus – Polyoma middle T (MMTV-PyMT) transgenic mouse model results in resistance to cisplatin or doxorubicin-induced apoptosis. Herein, we aim to establish the mechanism that is responsible for APC-mediated chemotherapeutic resistance. Our data demonstrate that MMTV-PyMT;*Apc*^*Min/+*^ cells have increased signal transducer and activator of transcription 3 (STAT3) activation. STAT3 can be constitutively activated in breast cancer, maintains the tumor initiating cell (TIC) population, and upregulates multidrug resistance protein 1 (MDR1). The activation of STAT3 in the MMTV-PyMT;*Apc*^*Min/+*^ model is independent of interleukin 6 (IL-6); however, enhanced EGFR expression in the MMTV-PyMT;*Apc*^*Min/+*^ cells may be responsible for the increased STAT3 activation. Inhibiting STAT3 with a small molecule inhibitor A69 in combination with doxorubicin, but not cisplatin, restores drug sensitivity. A69 also decreases doxorubicin enhanced MDR1 gene expression and the TIC population enhanced by loss of APC. In summary, these results have revealed the molecular mechanisms of APC loss in breast cancer that can guide future treatment plans to counteract chemotherapeutic resistance.

## INTRODUCTION

Breast cancer is the most commonly diagnosed cancer in women in the United States [1]. Despite the high rate of diagnosis and early detection, breast cancer is still the 2nd leading cause of cancer death in women in the United States [1]. One of the primary contributors to poor prognosis in breast cancer patients is chemotherapeutic resistance. Although the exact mechanisms leading to chemotherapeutic resistance have not been fully elucidated, it is known that molecular changes that affect signaling pathways can impact chemotherapeutic resistance. Chemotherapeutic resistance can be intrinsic or acquired and a number of cell signaling pathways can lead to resistance (reviewed by [2]). For our studies, we are focused on intrinsic chemotherapeutic resistance and determining the signaling pathways that are involved in this resistance. Although a number of theories have been proposed (reviewed by [3]), the most common cause of chemotherapeutic resistance is multidrug resistance driven by alterations in gene expression for ATP binding cassette efflux pumps (ABC transporters), which affect the rate of drug efflux from the tumor cells. Therefore, increased pump expression results in tumor cells being less responsive to chemotherapeutic agents.

Chemotherapy is often used to treat a variety of breast cancer subtypes either in a neoadjuvant or adjuvant fashion. The drugs used can vary and are often given in combination with different classes of chemotherapeutic agents. Doxorubicin alone or in combination with other chemotherapeutic agents is one of the most common treatments for women with triple negative breast cancer [4]. Cisplatin is also used to treat triple negative breast cancer [5, 6]. Our work focuses on determining the mechanism(s) involved in cisplatin and doxorubicin resistance incurred from loss of APC. Doxorubicin is an anthracyline that intercalates into the DNA and prevents cellular replication [7], whereas cisplatin is a platinum-based drug that forms DNA adducts (reviewed in [6]).

Loss of *Adenomatous Polyposis Coli* (APC) through mutation or hypermethylation occurs in up to 70% of sporadic breast cancer patients [8–10]. We previously reported that heterozygous *Apc* mutation in the Mouse Mammary Tumor Virus – Polyoma middle T (MMTV-PyMT) transgenic mouse model leads to enhanced tumorigenesis [11]. Based on the interaction of APC with its binding partners, we assessed the cellular response to cisplatin, paclitaxel, and doxorubicin [12], and we made the novel observation that APC loss led to doxorubicin and cisplatin resistance. Therefore, we focused on the effects of doxorubicin and cisplatin for these studies. Cells isolated from these tumors have increased expression of multidrug resistance protein 1 (MDR1) and tumor initiating cell (TIC) populations that may be responsible for the resistance to cisplatin or doxorubicin-mediated apoptosis [12]. Knowledge of the potential mechanism of APC-mediated chemotherapeutic resistance can be used to develop combination treatments to overcome this resistance. We previously demonstrated that synergism between cisplatin and Src or JNK inhibition restored cisplatin sensitivity in the MMTV-PyMT;*Apc*^*Min/+*^ cells; however, the same relationship was not evident with doxorubicin [12]. While doxorubicin treatment was not impacted by Src or JNK inhibition, doxorubicin enhanced MDR1 gene expression in MMTV-PyMT;*Apc*^*Min/+*^ cells [12]. These data point to divergent mechanisms of action between cisplatin and doxorubicin resistance in the absence of APC.

Signal transducer and activator of transcription 3 (STAT3) is a potential modulator of chemotherapeutic resistance in the model of APC loss in breast cancer. STAT3 is constitutively activated in triple negative breast cancer tissues and cells lines and helps maintain the population of TICs [13]. TICs have higher levels of ABC transporters including MDR1 compared to normal cells and can impact chemotherapeutic resistance [14]. TICs can be identified by over-expression of activated STAT3 [15, 16]. STAT3 can upregulate MDR1 gene expression as well [17–19]. Our data indicate that doxorubicin resistance may occur through MDR1 since MDR1 expression is augmented by treatment with doxorubicin in MMTV-PyMT;*Apc*^*Min/+*^ cells [12]. Doxorubicin is one chemotherapeutic agent that is effluxed by MDR1 (reviewed by [20]).

Understanding the molecular mechanisms downstream of APC loss in breast cancer will be important in future treatment plans, especially the development of individualized treatment plans. Patients can become resistant to many of the commonly used chemotherapeutic agents. We report here that inhibition of STAT3 with the small molecule inhibitor, A69, restores sensitivity to doxorubicin, but not cisplatin, in MMTV-PyMT;*Apc*^*Min/+*^ cells.

## RESULTS

**Figure 1:**
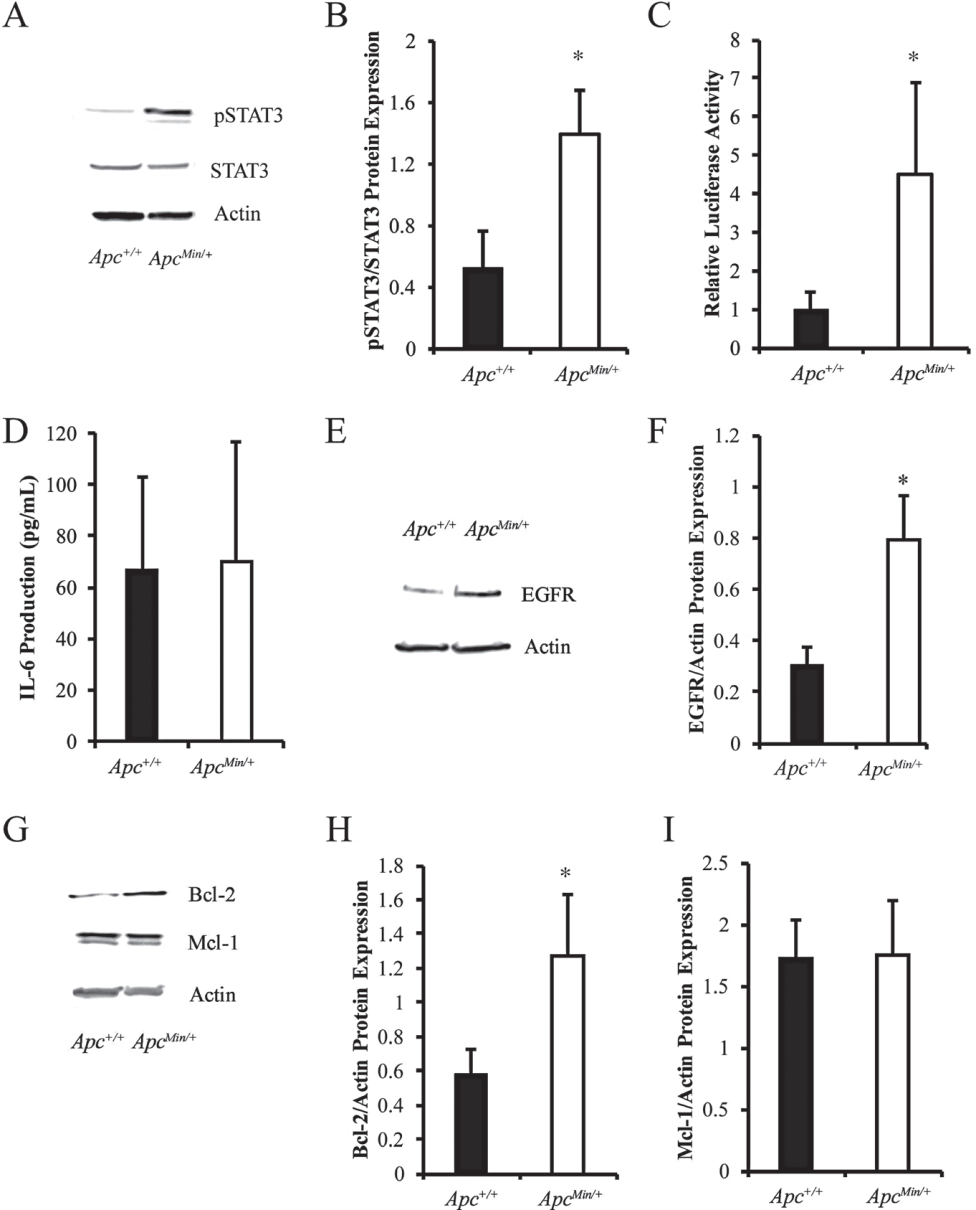
Loss of APC leads to increased activation of STAT3 (**A**) Western blot analysis demonstrates that MMTV-PyMT;*Apc*^*Min/+*^ cells have increased pSTAT3 but similar levels of total STAT3. (**B**) Quantification of pSTAT3/STAT3 in MMTV-PyMT;*Apc*^*Min/+*^ vs MMTV-PyMT;*Apc*^*+/+*^ cells shows a significant increase in activated STAT3. (**C**) Western blot results were confirmed using a dual luciferase reporter assay. Cells were transfected with STAT3 and pRL-TK reporter plasmids for 24 hours and luciferase activity was measured. (**D**) IL-6 production was measured in media using ELISA, and no difference was observed between MMTV-PyMT;*Apc*^*+/+*^ and MMTV-PyMT;*Apc*^*Min/+*^ cells. (**E**) Representative western blots for total EGFR show an increase in MMTV-PyMT;*Apc*^*Min/+*^ compared to MMTV-PyMT;*Apc*^*+/+*^ cells. (**F**) Quantification of EGFR normalized to actin shows that MMTV-PyMT;*Apc*^*Min/+*^ cells express higher levels of EGFR compared to MMTV-PyMT;*Apc*^*+/+*^ cells. (**G**) Representative western blots for the anti-apoptotic proteins, Bcl-2 and Mcl-1, and quantification (**H**) shows that Bcl-2 is increased in MMTV-PyMT;*Apc*^*Min/+*^ cells compared to MMTV-PyMT;*Apc*^*+/+*^ cells, while Mcl-1 (**I**) is unchanged. Actin was used as a loading control for all western blot experiments. Each experiment was repeated 3 times and data are shown as means +/- SD; *P < 0.05 comparing MMTV-PyMT;*Apc*^*Min/+*^ to MMTV-PyMT;*Apc*^*+/+*^ cells.

We previously demonstrated that loss of APC in the MMTV-PyMT mouse model results in resistance to cisplatin and doxorubicin-induced apoptosis, increased MDR1 expression, and increased the TIC population [12]. Given that increased MDR1 and TICs are known mechanisms of chemotherapeutic resistance and are impacted by STAT3 activation, we sought to determine whether STAT3 was involved in APC-mediated chemotherapeutic resistance. The MMTV-PyMT;*Apc*^*Min/+*^ cells have increased levels of phosphorylated STAT3 (pSTAT3) protein, with no difference in total STAT3 (Figure 1A and 1B). The functionality of the increased pSTAT3 was confirmed using a dual-luciferase reporter assay to show that MMTV-PyMT;*Apc*^*Min/+*^ cells have increased transcriptional activity of STAT3 (Figure 1C). We sought to understand how APC loss results in aberrant STAT3 activation. As STAT3 is often activated through IL-6 dependent pathways [13, 21, 22], we used a mouse specific IL-6 ELISA and demonstrated no difference in IL-6 production between the two cell lines (Figure 1D). In addition to IL-6-mediated STAT3 phosphorylation, aberrant EGFR signaling can also increase STAT3 activation [23, 24]. Investigation of EGFR demonstrated that MMTV-PyMT;*Apc*^*Min/+*^ cells have increased EGFR expression (Figure 1E and 1F), suggesting this as a possible link between APC loss and STAT3 activation. Based on these data, we speculate that EGFR, not IL6, is responsible for STAT3 activation in the MMTV-PyMT;*Apc*^*Min/+*^ cells. To understand how hyperactivation of STAT3 may impact chemoresistance, we looked at genes under transcriptional control by STAT3, including Mcl-1 and Bcl-2, which are pro-survival proteins in the apoptosis cascade. Given the impact of APC loss on the apoptotic response after chemotherapy treatment, Bcl-2 and Mcl-1 protein levels were assessed. We show that the MMTV-PyMT;*Apc*^*Min/+*^ cells have increased expression of Bcl-2 (Figure 1G and 1H), but not Mcl-1 (Figure 1G and 1I).

**Figure 2:**
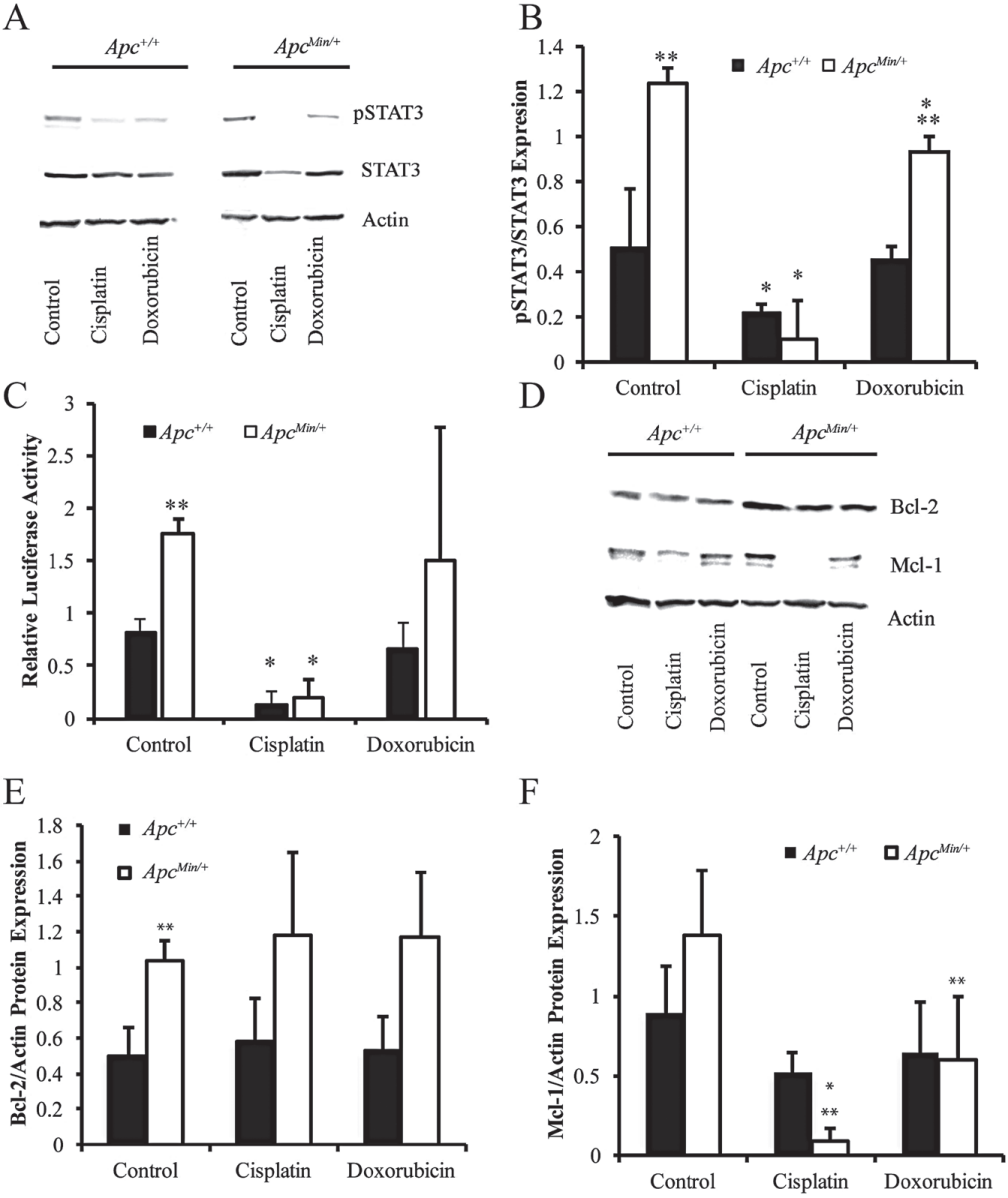
Chemotherapeutic treatments affect activation of STAT3 (**A**) Representative western blots showing the changes in pSTAT3 and STAT3 in MMTV-PyMT;*Apc*^*Min/+*^ vs MMTV-PyMT;*Apc*^*+/+*^ cells treated with cisplatin or doxorubicin for 24 hours. (**B**) Quantification of westerns blots shows that cisplatin nearly eliminates pSTAT3 in both cell lines. (**C**) Dual luciferase reporter assays measured STAT3 activation after treatment with cisplatin and doxorubicin and confirmed that cisplatin decreased STAT3 activity in both cells lines. (**D**) Representative western blots for Mcl-1 and Bcl-2 after 24-hour treatment with cisplatin and doxorubicin. (**E**) Quantification of western blots shows that chemotherapeutic agents do not affect Bcl-2 at the protein level. (**F**) Western blot quantification demonstrates that both cisplatin and doxorubicin decrease Mcl-1 in MMTV-PyMT;*Apc*^*Min/+*^ cells. Each experiment was repeated 3 times and data are shown as means +/- SD; *P < 0.05 compared to solvent control, **P < 0.05 comparing MMTV-PyMT;*Apc*^*Min/+*^ to MMTV-PyMT;*Apc*^*+/+*^ cells

Next, we aimed to understand how treatment with cisplatin and doxorubicin affects activation of STAT3 to determine whether this would impact chemotherapeutic resistance. Cisplatin treatment for 24 hours significantly decreased pSTAT3 in both cell lines (Figure 2A and 2B). Doxorubicin also slightly decreased pSTAT3 expression selectively in the MMTV-PyMT;*Apc*^*Min/+*^ cells (Figure 2A and 2B). Similar results were obtained with the STAT3 reporter assay confirming a loss of STAT3 transcriptional activity after cisplatin treatment in both cell lines (Figure 2C). Treatment with chemotherapeutic agents had no effect on Bcl-2 protein expression (Figure 2D and 2E). Interestingly, cisplatin treatment, and doxorubicin to a lesser extent, for 24 hours decreased Mcl-1 protein expression similar to the effect on pSTAT3 (Figure 2D and 2F).

**Figure 3:**
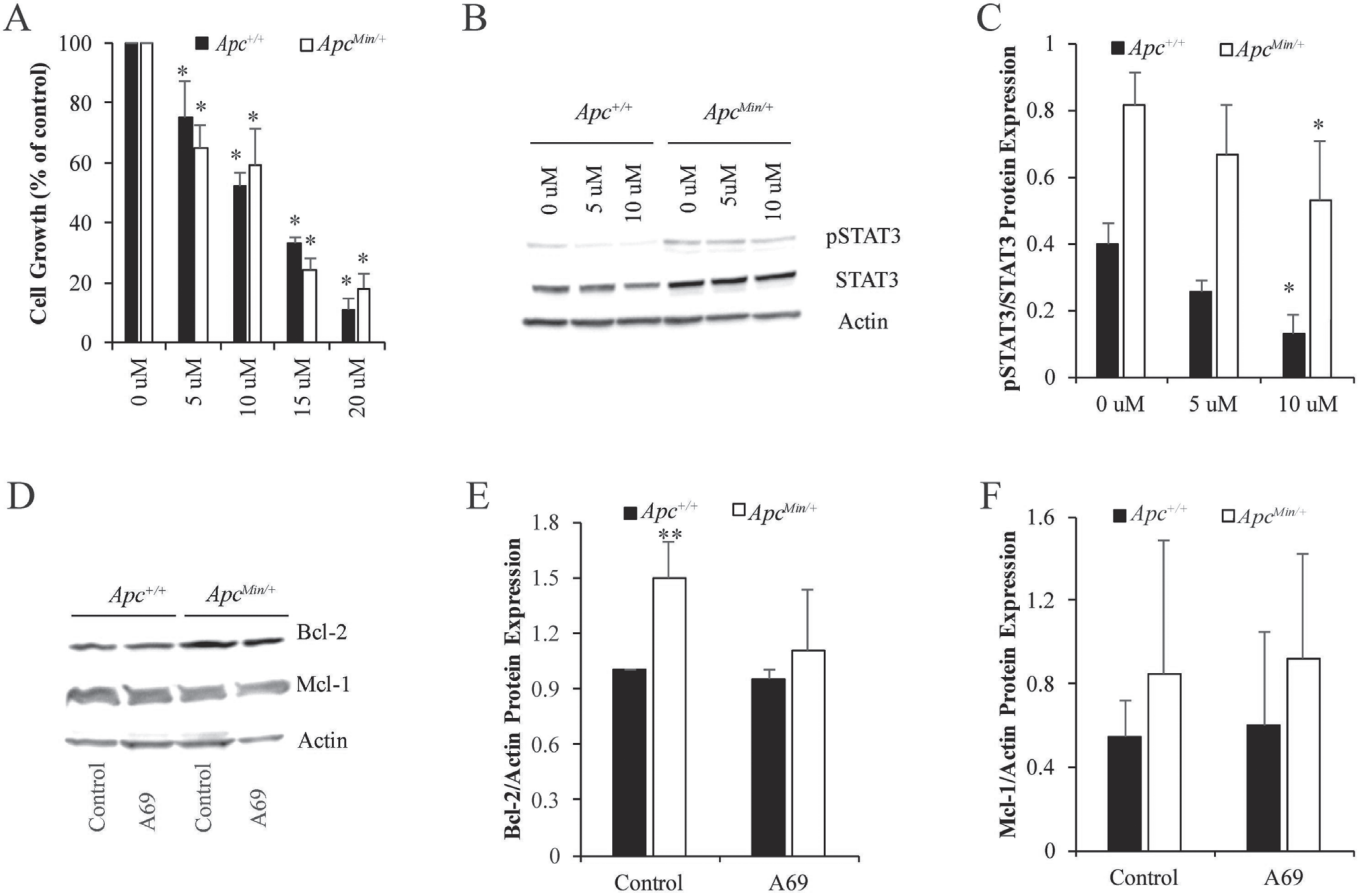
STAT3 inhibition in MMTV-PyMT;*Apc*^*Min*/+^ and MMTV-PyMT;*Apc*^+/+^ cells (**A**) Cell growth was measured for 24 hours with treatment 0–20 μM A69, a small molecule inhibitor of STAT3. Data are shown as the percent of cells compared to the untreated control (set to 100% for each cell line individually). (**B**) Representative western blots for pSTAT3/STAT3 after treatment with 0–10 μM of A69 for 24 hours. (**C**) Quantification of pSTAT3/STAT3 western blots show that 10 μM modestly decreases STAT3 phosphorylation in both cell lines. 10 μM was selected as the dose for A69 for future studies. (**D**) Representative western blots for Mcl-1 and Bcl-2 after 24-hour treatment with 10 μM A69. (**E**–**F**) Quantification of Bcl-2 and Mcl-1 western blots demonstrates that A69 does not affect Bcl-2 or Mcl-1 expression. Each experiment was repeated at least 3 times and data are shown as means +/- SD; *P < 0.05 compared to solvent control, **P < 0.05 comparing MMTV-PyMT; *Apc*^*Min/+*^ to MMTV-PyMT; *Apc*^*+/+*^ cells.

Recently, a group of selective small molecule inhibitors targeting the DNA-binding domain of STAT3 has been identified and shown to inhibit cell proliferation and migration *in vitro* and tumor development *in vivo* [25, 26]. Here, we used one of these STAT3 inhibitors, A69, to determine the impact of STAT3 inhibition on growth of MMTV-PyMT;*Apc*^*Min/+*^ and MMTV-PyMT;*Apc*^*+/+*^ cells. Initial concentrations of A69 were determined based on previous studies in multiple cell lines [25, 26]. Cell growth assays determined that A69 (10 μM for 24 hours) was effective in reducing cell numbers by 40–50% (Figure 3A). Western blot analysis demonstrated that A69 (10 uM) resulted in a slight decrease in STAT3 activation at 24 hours (Figure 3B and 3C). To understand whether the STAT3 activity was directly responsible for Bcl-2 and Mcl-1, we performed western blots on cell lysates treated with A69 for 24 hours. Treatment with A69 for 24 hours had no effect on Bcl-2 or Mcl-1 protein expression (Figure 3D–3F).

**Figure 4:**
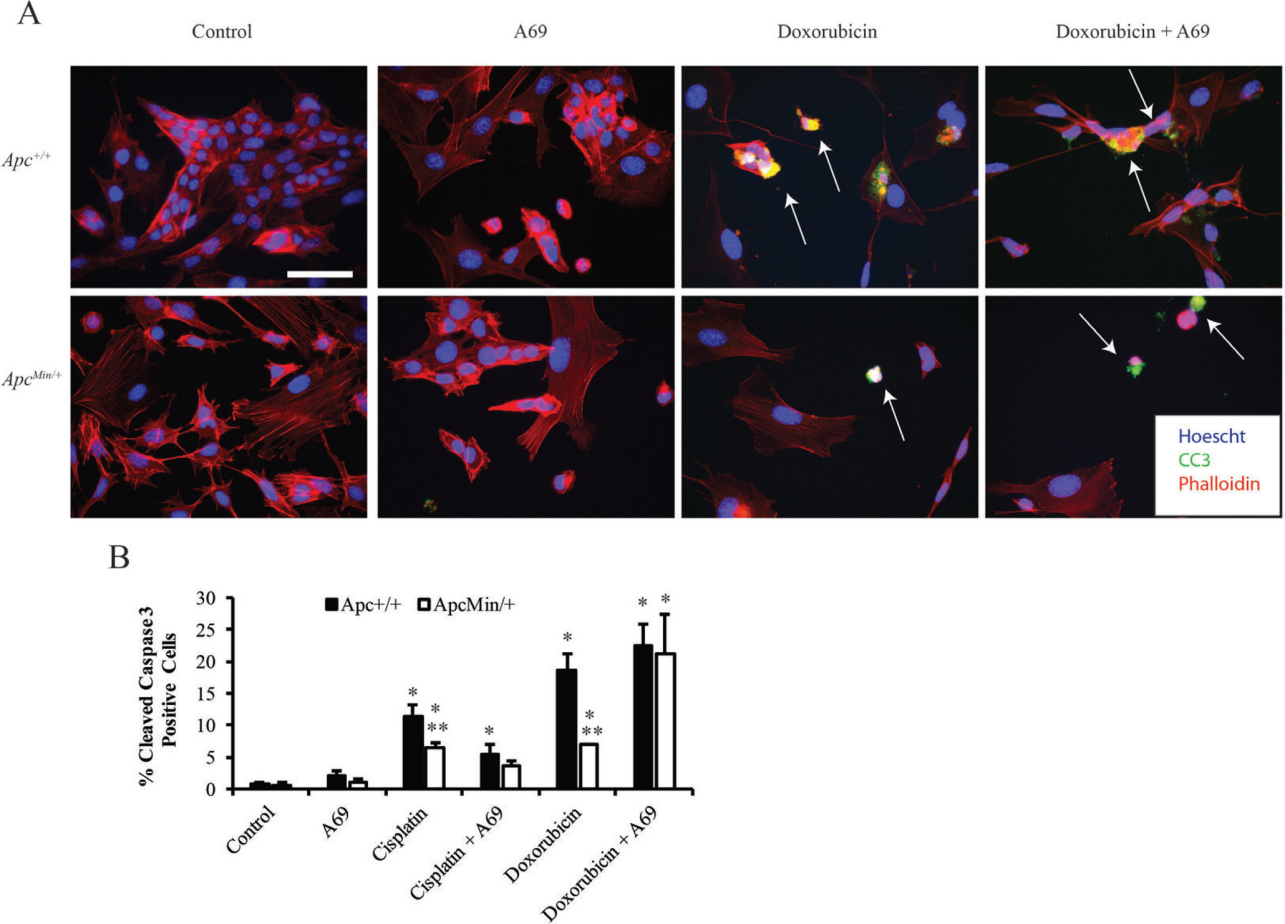
Effects of chemotherapeutic agents on apoptosis (**A**) Representative immunofluorescence (IF) images for MMTV-PyMT;*Apc*^*Min/+*^ cells treated with A69, doxorubicin, or the combination of A69 and doxorubicin. White arrows depict cleaved caspase 3 (CC3) positive cells, and the scale bar is 200 μM. (**B**) Quantification of the percent of CC3 positive cells after 24-hour treatment with single agents or combination of A69 with either cisplatin or doxorubicin. A69 alone does not cause a significant increase in apoptosis. Treatment with A69 in combination with doxorubicin restores sensitivity in MMTV-PyMT;*Apc*^*Min/+*^ cells. The experiment was repeated 3 times, at least 150 cells were counted per condition in each experiment, and data are shown as means +/- SD; *P < 0.05 when compared to solvent control, **P < 0.05 comparing MMTV-PyMT;*Apc*^*Min/+*^ to MMTV-PyMT;*Apc*^*+/+*^ cells.

Given that we previously demonstrated that MMTV-PyMT;*Apc*^*Min/+*^ cells were resistant to cisplatin and doxorubicin [12], we next wanted to understand whether inhibiting STAT3 would restore the apoptotic response. Using cleaved caspase 3 (CC3) immunofluorescence (IF), we showed that A69 alone does not induce a robust increase in apoptosis (Figure 4A and 4B). A69 sensitized MMTV-PyMT;*Apc*^*Min/+*^ cells to doxorubicin (Figure 4A and 4B), but had no impact on the response to cisplatin in the MMTV-PyMT;*Apc*^*Min/+*^ cells (Figure 4B). The combination of doxorubicin and A69 synergistically increased the apoptotic response in the MMTV-PyMT;*Apc*^*Min/+*^ cells (Figure 4A and 4B, *p* < 0.0001). These data indicate that STAT3 inhibition specifically restores sensitivity to doxorubicin in the MMTV-PyMT;*Apc*^*Min/+*^ cells.

**Figure 5:**
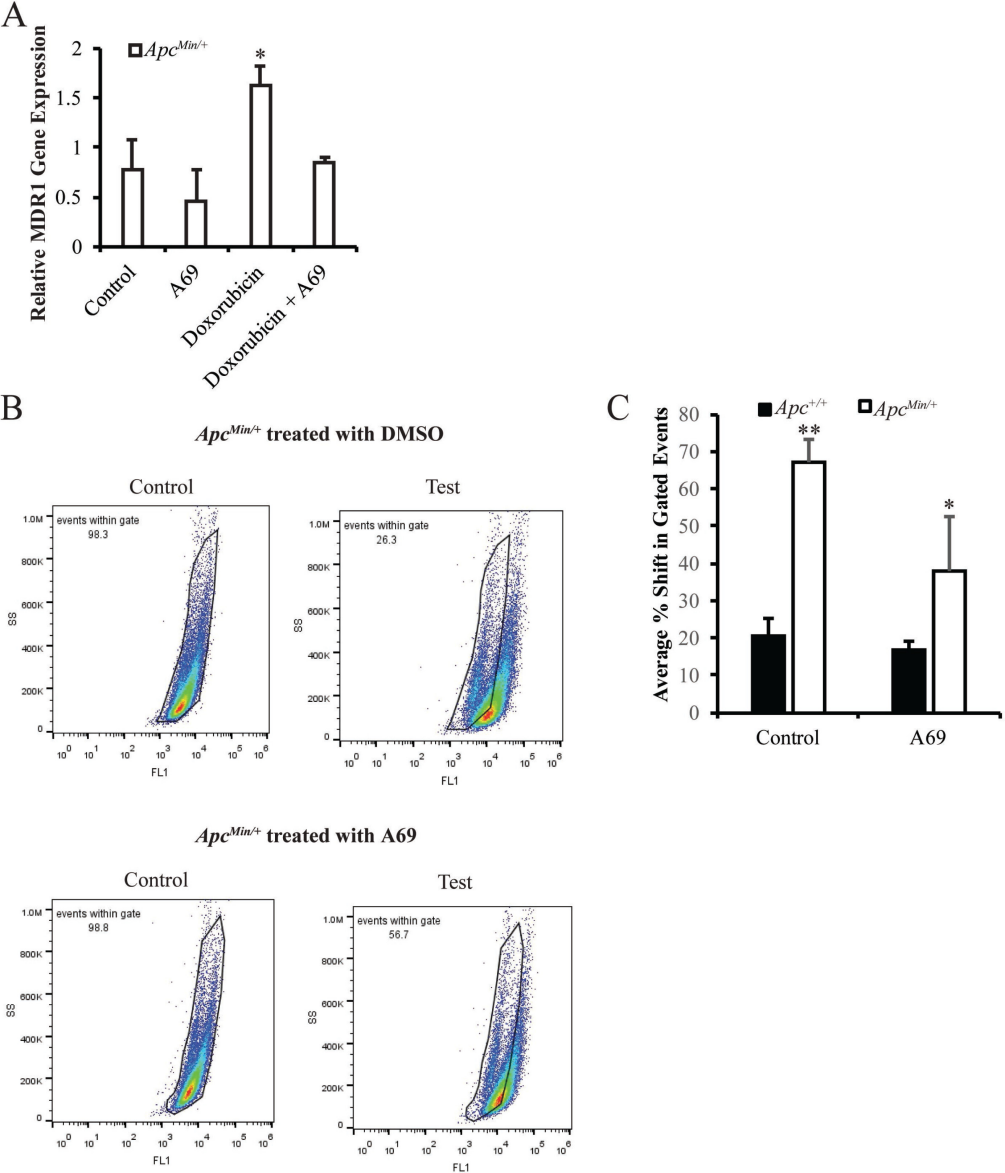
Effects of A69 on MDR1 gene expression and the TIC population (**A**) The doxorubicin-induced MDR1 gene expression in MMTV-PyMT;*Apc*^*Min/+*^ cells is inhibited by A69. Expression is normalized to 18s rRNA. (**B**) Representative FACS analysis of MMTV-PyMT;*Apc*^*Min/+*^ cells treated with solvent control or A69. (**C**) Quantification of Aldefluor assay in MMTV-PyMT;*Apc*^*Min/+*^ and MMTV-PyMT;*Apc*^*+/+*^ cells treated with solvent control or A69. Data are shown as the shift in gated events between test and control samples. Control samples were treated with an inhibitor DEAB. Each experiment was repeated 3 times and data are shown as means +/- SD; *P < 0.05 compared to solvent control, **P < 0.05 when comparing MMTV-PyMT;*Apc*^*Min/+*^ vs MMTV-PyMT;*Apc*^*+/+*^ cells.

To further investigate how increased activation of STAT3 results in doxorubicin resistance in the MMTV-PyMT;*Apc*^*Min/+*^ cells, we focused the remainder of our studies on STAT3-induced MDR1 and TIC population. Based on the links between STAT3 and these two pathways [19], and having previously made the observation that MMTV-PyMT;*Apc*^*Min/+*^ cells have increased MDR1 expression that is enhanced by treatment with doxorubicin [12], we performed qRT-PCR to determine the effect of A69 on MDR1 gene expression in MMTV-PyMT;*Apc*^*Min/+*^ cells. While we found no effect of A69 alone, when treated in combination with doxorubicin, A69 blocked the ability of doxorubicin to increase MDR1 expression (Figure 5A). There were no effects of A69 on MDR1 gene expression in the MMTV-PyMT;*Apc*^*+/+*^ cells (data not shown and [12]). To understand the relationship between the increased activation of STAT3 and the increased TIC population that we observed in the MMTV-PyMT;*Apc*^*Min/+*^ cells [12], Aldefluor assays were performed. As expected, there was no effect of A69 on the TIC population of the MMTV-PyMT;*Apc*^*+/+*^ cells. A69 treatment significantly decreased the TIC population in the MMTV-PyMT;*Apc*^*Min/+*^ cells (Figure 5B and 5C). However, the TIC population was not reduced to that of the MMTV-PyMT;*Apc*^*+/+*^ cells, suggesting that STAT3 acts in combination with other signaling modalities to enhance the TIC population.

## DISCUSSION

We have previously demonstrated that loss of APC in the MMTV-PyMT mouse transgenic model results in resistance to both cisplatin and doxorubicin that is independent of Wnt/β-catenin activation [12]. The molecular mechanisms resulting in chemotherapeutic resistance mediated by APC loss are not well understood. We have established a potential pathway that can be targeted in breast cancer patients to restore sensitivity to doxorubicin. Loss of APC in the MMTV-PyMT mouse model increases activation of STAT3, which is constitutively activated in approximately 50% of breast cancer cell lines and tumor samples [13].

We used Mcl-1 and Bcl-2 expression as an indicator of downstream activity of STAT3 since both proteins are involved in the apoptotic response. Inhibition of STAT3 by both curcumin and its analogue hydrazinocurcumin blocks protein expression of both Mcl-1 and Bcl-2 in MDA-MB-231 and MCF-7 breast cancer cells [27]. Although we did not note a difference in Mcl-1 or Bcl-2 protein expression after treatment with A69, this may be due to alternate components of the STAT3 signaling pathway. We also showed that treatment with cisplatin reduces Mcl-1 expression, which may be due to the loss of STAT3 activation. These results are similar to those in renal tubular epithelial cells where treatment with cisplatin affected Mcl-1 expression but not Bcl-2 expression [28].

Since inhibition of STAT3 restores sensitivity to doxorubicin in the MMTV-PyMT;*Apc*^*Min/+*^ mouse model, we were interested in understanding how this restoration occurred. We focused on two specific mechanisms, MDR1 expression and TIC population, that we have shown to possibly be involved in chemotherapeutic resistance in the MMTV-PyMT;*Apc*^*Min/+*^ cells [12]. Others have also demonstrated that inhibition of STAT3 decreases the TIC population in breast cancer cells [15]. A69 reduced the TIC population in MMTV-PyMT;*Apc*^*Min/+*^ cells but did not restore levels to that of the MMTV-PyMT;*Apc*^*+/+*^ cells, suggesting alternate factors associated with *Apc* loss that enhance the TIC population. A number of signaling pathways besides STAT3 can enhance TICs in breast cancer including NOTCH signaling, Hedgehog signaling, and integrins (reviewed in [29]). We also found that A69 prevents doxorubicin from enhancing MDR1 gene expression, which may be responsible for the intrinsic doxorubicin resistance observed in the MMTV-PyMT;*Apc*^*Min/+*^ cells. As cisplatin is not effluxed by MDR1 [20], we hypothesize this is the primary reason A69 could not restore cisplatin sensitivity. Interestingly, STAT3 can also play a role in modulating DNA damage pathways [30], which could contribute to the STAT3-mediated doxorubicin resistance in MMTV-PyMT;*Apc*^*Min/+*^ cells. The role of STAT3 in DNA damage has been highlighted in fibrosarcoma, where cells with low STAT3 levels have decreased ATM-Chk1 and ATM-Chk2 via transcriptional regulation of MDC1 [30].

Our initial hypothesis aimed to understand whether IL-6 was responsible for APC-mediated STAT3 activation. IL-6 commonly activates STAT3 in breast cancer models (reviewed in [31, 32]). IL-6 can be produced in an autocrine fashion in both breast [17], and ovarian [33], cancer and therefore is an important step in tumorigenesis (reviewed in [32]). Our results demonstrated that IL-6 is not responsible for the activation of STAT3 in MMTV-PyMT;*Apc*^*Min/+*^ cells. In addition to IL-6, STAT3 can be activated by EGFR in many cancer types (reviewed in [31]). There is also a strong association between nuclear STAT3 and EGFR expression in breast cancer samples and this EGFR/STAT3 relationship enhances tumorigenesis [34]. STAT3 is also an important player in the cross-talk that occurs with EGFR signaling [35]. Studies in *Drosophila* show that loss of *Apc* leads to activation of both STAT3 and EGFR [36]. In *Apc*^*Min/+*^ mice there is increased EGFR activity, demonstrating that the increased EGFR expression observed here may be directly linked to mutant *Apc* [37]. Previous literature demonstrated that EGFR recycling could be impacted by cell stress, suggesting a possible link between APC-mediated aberrant signaling and EGFR recycling [38]. Future work will dissect how APC loss leads to increased EGFR expression, focusing on specific post-translational modifications and the recruitment and recycling of EGFR.

Chemotherapeutic resistance can also be caused by evading apoptosis and changing DNA repair mechanisms [3]. Future work will focus on these pathways as potential targets for resolving APC-mediated cisplatin resistance. We found that cisplatin treatment decreased activation of STAT3; however, this did not lead to cell death indicating activation of other signaling pathways allowing the MMTV-PyMT;*Apc*^*Min/+*^ cells to evade cisplatin-induced apoptosis. Previously, we showed that combination treatments with inhibitors for JNK (SP600125) and Src (PP2) restore sensitivity of MMTV-PyMT;*Apc*^*Min/+*^ cells to cisplatin [12]. Thus future work will also delineate how these pathways are involved in APC-mediated cisplatin resistance.

**Figure 6:**
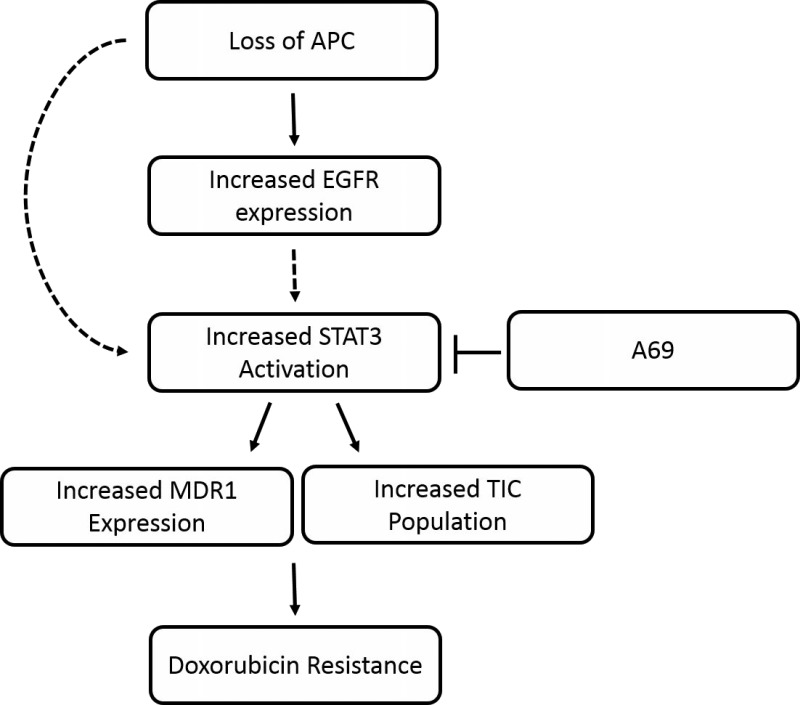
Schematic of APC-mediated doxorubicin resistance Predicted model of how APC loss results in resistance to doxorubicin. We have shown that APC loss increases the phosphorylation of EGFR and STAT3. In addition, blocking STAT3 with A69 blocks the APC-mediated effects on MDR1 expression and the TIC population. Finally, A69 is able to restore sensitivity to doxorubicin in the *Apc*^*Min/+*^ cells. The dashed line from EGFR to STAT3 indicates a potential regulation that we are currently investigating. The dashed line from “loss of APC” to STAT3 suggests that there may be other signaling modalities that we have yet to uncover.

In conclusion, our results demonstrate that the STAT3 signaling pathway is important in the development of APC-mediated doxorubicin resistance but not cisplatin resistance. The effects may be due to EGFR upstream of STAT3 or changes in MDR1 and the TIC population downstream of STAT3 (Figure 6). These results will need to be confirmed using *in vivo* mouse models and may lead to promising combination treatments to overcome APC-mediated doxorubicin resistance.

## MATERIALS AND METHODS

### Cell culture and drug treatment

MMTV-PyMT;*Apc*^*+/+*^ and MMTV-PyMT;*Apc*^*Min/+*^ cells were isolated from primary tumors from the mouse mammary gland [11] and grown in RPMI 1640 media supplemented with 10% fetal bovine serum, 1% penicillin/streptomycin, and 1:5000 plasmocin (In*vivo*gen, San Diego, CA). All cells were routinely passaged using 0.25% trypsin/EDTA and maintained at 37°C with 5% CO_2_. Experiments were performed with cells between passage 10–20. Cells were treated for 24 hours with each chemotherapeutic agent or solvent control: doxorubicin (500 nM MP Biomedicals, LLC, Santa Ana, CA) or cisplatin (16 μM; cis-Diammineplatinum (III) dichloride, Sigma-Aldrich) as described previously [12]. STAT3 DNA binding was blocked by 24-hour treatment with the small molecule inhibitor A69 [25, 26].

### Cell growth assays

50,000 cells were plated per well in 24-well plates in duplicate. 24 hours later cells were treated with 0–20 μM A69. 24 hours post-treatment, adherent cells were trypsinized and counted. Data are normalized to the solvent control for each cell line.

### Western blots

Total protein was isolated using a phosphatase inhibitor enhanced lysis buffer (20 mM Tris-HCl, 150 mM NaCl, 1% Triton-X, 0.5% NP-40, 50 mM NaF, 1 mM Na_3_VO_4_, 5 mM Sodium pyrophosphate, 0.2 mM PMSF, 1x protease inhibitor cocktail (Fisher), and 1x phosphatase inhibitor cocktail 2 (Sigma)). Protein concentration was measured using a BCA assay. 20–25 µg of protein was separated by SDS-PAGE (10% gel), and transferred onto Immobilon-P membrane (Millipore). Following protein transfer, membranes were blocked for 1 hour at room temperature in 5% non-fat dry milk in 1XTBS with 0.1% Tween (TBS-T). Blots were incubated with primary antibody diluted 1:1000 in 1% bovine serum albumin in TBS-T overnight at 4°C: STAT3 (#12640, Cell Signaling), pSTAT3 (#9145, Tyrosine 705, Cell Signaling), Mcl-1 (#5453, Cell Signaling) Bcl-2 (#2870, Cell signaling) or EGFR (#4267, Cell Signaling). Membranes were incubated in secondary antibody (anti-rabbit IgG-HRP, 1:1000 in 1% bovine serum albumin in TBS-T) at room temperature for 1 hour. Blots were developed with Clarity ECL reagent (Bio-Rad) and a ChemiDoc MP Imaging System (Bio-Rad). Blots were reprobed for β-actin (1:25,000, 1% BSA in TBS-T, Sigma) for 1 hour at room temperature followed by anti-mouse IgG-HRP (1:2000 in 1% bovine serum albumin in TBS-T) for 1 hour at room temperature to verify equal protein loading. Densitometry quantification was performed using ImageJ software (NIH). Blots are representative of three replicates.

### Reporter assays

75,000 cells were plated in 24 well plates in triplicate and 24 hours later cells were transfected with lipofectamine 2000 (6 µl, Life Technologies) and 6 µg of STAT3 Plasmid (Promega; pGL4.47(luc2P/SIE/Hygro)) and pRL-TK (for transfection efficiency, Promega). When cells were also treated with chemotherapeutic agents, treatments were done at the time of transfection. After 24 hours, lysates were collected and luciferase activity was measured using the Dual Luciferase Assay Kits (Promega) according to manufacturer’s recommendations. A SpectraMax M3 plate reader (Molecular Devices, Sunnyvale, CA) was used to measure luciferase activity. We normalized firefly luciferase values to renilla luciferase values to account for differences in cell transfection.

### Immunofluorescence

Apoptosis was determined using cleaved caspase 3 IF as previously described [12]. Briefly 40,000 cells were seeded in 12 well plates on glass coverslips in triplicate. 24 hours later cells were treated with chemotherapeutic agents with or without A69 for 24 hours. After treatment, cells were fixed in 3.7% formaldehyde for 15 minutes and permeabilized in 0.3% Triton X-100 for 15 minutes. Antibodies were diluted in blocking buffer that consisted of 0.2% non-fat dry milk, 2% Bovine Serum Albumin and 0.3% Triton X-100 in phosphate buffered saline (PBS). Primary anti-cleaved caspase 3 rabbit monoclonal antibody (1:400, Cell Signaling Technology, Danvers, MA) was applied to cells for 1 hour at 37°C. Following washes in PBS, samples were incubated in goat-anti-rabbit Alexa Fluor 488 (1:1000, Life Technologies, Carlsbad, CA) and Alexa 555 conjugated Phalloidin (1:200, Life Technologies) to visualize F-actin. Slides were mounted with Fluoromount G with Hoescht (Sigma) to label cell nuclei. The percentage of positive cells was determined for each assay with at least 150 cells being counted per condition using the counting feature on an Evos Xl core microscope. Each assay was run in triplicate and repeated three times. Representative images were taken on Zeiss Axio A1 Microscope with an AxioCam MRc digital camera.

### RNA isolation and RT-PCR

MMTV-PyMT;*Apc*^*+/+*^ and MMTV-PyMT;*Apc*^*Min/+*^ cells were seeded at 8**×**10^5^ cells per 10 cm plate for 24 hours then treated with chemotherapy drugs and/or A69 as above for 24 hours. RNA was isolated using Tri Reagent (Molecular Research Center, Cincinnati, OH). cDNA synthesis was performed with iScript from 1 μg total RNA (Bio-Rad Laboratories, Hercules, CA). Real-time RT-PCR was performed using Power SYBR Green master mix (Applied Biosystems, Foster City, CA), 50 ng of cDNA, and 7.5 µM (or 3.75 µM for 18s) of each primer. Primers were the same as used previously for 18s rRNA (reference gene) and MDR1 [12]. Primers were 18s F 5′-GGCGGCTTGGTGACTCTAGAT-3’, 18s R 5′-CTTCCTTGGATGTGGTAGCCG-3’ MDR1 F 5′-CATTGGTGTGGTGAGTCAGC-3’, MDR1 R 5′-CTCTCTCTCCAACCAGGGTC-3’. The amplification program included 2 initial steps at 50° C for 2 minutes and 95° C for 10 minutes followed by 40 cycles of 95° C for 15 seconds and 60°C for 1 minute followed by generation of a melt curve (CFX Connect 96 thermal cycler, Bio-Rad). Samples were run in duplicate and the experiment was replicated 3 times. All MMTV-PyMT;*Apc*^*Min/+*^ samples plus the MMTV-PyMT;*Apc*^*+/+*^ solvent control were run on one plate for each run and MMTV-PyMT;*Apc*^*+/+*^ cells plus the MMTV-PyMT;*Apc*^*Min/+*^ solvent control were run on one plate. This allowed for comparisons between the MMTV-PyMT;*Apc*^*+/+*^ control and MMTV-PyMT;*Apc*^*Min/+*^ cells with all combinations of treatments and vice versa.

### Aldefluor assay

To determine the population of TICs, we performed Aldefluor assays (Stem Cell Technologies, Vancouver, British Colombia) according to manufacturer’s recommendations and as previously described [12]. Briefly, 1 × 10^6^ cells were plated in a 10 cm dish overnight prior to 24-hour treatment with A69 (10 μM). After A69 treatment, 2 × 10^5^ cells were suspended in Aldefluor assay buffer containing ALDH substrate (Bodipy-Aminoacetaldehyde or BAAA), which served as the “test” sample. As a control, half of this sample was moved to a second tube where diethylaminobenzaldehyde (DEAB), a specific ALDH1 enzyme inhibitor was also added. Both samples were incubated for 60 minutes at 37°C. The fluorescent ALDH-expressing cells were detected in the green channel (515–535 nm) of a Cytotomics FC 500 (Beckman Coulter, Brea, CA) flow cytometer. Data were analyzed using FlowJo Flow Cytometry Data Analysis Software (Tree Star, Ashland, OR). The percent shift between gated events in the test versus control samples was calculated to give the relative TIC population.

### Interleukin 6 ELISA

The production of the cytokine IL-6 was measured in media in MMTV-PyMT;*Apc*^*Min/+*^ and MMTV-PyMT;*Apc*^*+/+*^ cells. 1x10^6^ cells were plated in a 10cm plate, and after 24 hours, media was removed and plates were washed with PBS. New media was added to plates and cells were allowed to grow for 24 hours prior to media collection and stored at –80°C. Enzyme linked immunosorbent assays (ELISA) specific for mouse IL-6 were run according the manufacturer’s instructions (Enzo Life Sciences, Farmingdale, NY). Chemiluminescence was measured on a SpectraMax M3 plate reader (Molecular Devices). Samples were run in duplicate and a 7-point standard curve was used to calculate concentration in pg/mL.

### Statistical analysis

Student’s *t*-tests were used for any analysis that included only comparisons for MMTV-PyMT;*Apc*^*+/+*^ versus MMTV-PyMT;*Apc*^*Min/+*^ cells. For all other analyses, a two-way ANOVA was performed using the LS Means statement with the Proc GLM platform of SAS University Edition (Cary, NC) for all pre-planned comparisons. A *p*-value < 0.05 was considered significant.

## Abbreviations

APC: Adenomatous Polyposis Coli
BAAA: Bodipy-Aminoacetaldehyde
CC3: Cleaved caspase 3
DEAB: Diethylaminobenzaldehyde
ELISA: Enzyme Linked Immunosorbent Assays
IF: Immunofluorescence
IL-6: Interleukin 6
MDR1: Multidrug Resistance Protein 1
MMTV-PyMT: Mouse Mammary Tumor Virus – Polyoma middle T
pSTAT3: phosphorylated STAT3
STAT3: Signal Transducer and Activator of Transcription 3
TIC: Tumor Initiating Cell
